# Dietary Supplementation of L-Arginine and N-Carbamylglutamate Attenuated the Hepatic Inflammatory Response and Apoptosis in Suckling Lambs with Intrauterine Growth Retardation

**DOI:** 10.1155/2020/2453537

**Published:** 2020-04-04

**Authors:** Hao Zhang, Yaotian Fan, Mabrouk Elsabagh, Shuang Guo, Mengzhi Wang, Honghua Jiang

**Affiliations:** ^1^Laboratory of Metabolic Manipulation of Herbivorous Animal Nutrition, College of Animal Science and Technology, Yangzhou University, Yangzhou 225009, China; ^2^Joint International Research Laboratory of Agriculture and Agri-Product Safety, The Ministry of Education of China, Yangzhou University, Yangzhou 225009, China; ^3^Department of Animal Production and Technology, Faculty of Agricultural Sciences and Technologies, Niğde Ömer Halisdemir University, Nigde 51240, Turkey; ^4^Department of Nutrition and Clinical Nutrition, Faculty of Veterinary Medicine, Kafrelsheikh University, Kafrelsheikh 33516, Egypt; ^5^Department of Pediatrics, Northern Jiangsu People's Hospital, Clinical Medical College, Yangzhou University, Yangzhou 225001, China

## Abstract

L-arginine (Arg) is a semiessential amino acid with several physiological functions. N-Carbamylglutamate (NCG) can promote the synthesis of endogenous Arg in mammals. However, the roles of Arg or NCG on hepatic inflammation and apoptosis in suckling lambs suffering from intrauterine growth restriction (IUGR) are still unclear. The current work is aimed at examining the effects of dietary Arg and NCG on inflammatory and hepatocyte apoptosis in IUGR suckling lambs. On day 7 after birth, 48 newborn Hu lambs were selected from a cohort of 432 twin lambs. Normal-birthweight and IUGR Hu lambs were allocated randomly (*n* = 12/group) to control (CON), IUGR, IUGR+1% Arg, or IUGR+0.1% NCG groups. Lambs were fed for 21 days from 7 to 28 days old. Compared with CON lambs, relative protein 53 (P53), apoptosis antigen 1 (Fas), Bcl-2-associated X protein (Bax), caspase-3, cytochrome C, tumor necrosis factor alpha (TNF-*α*), nuclear factor kappa-B (NF-*κ*B) p65, and NF-*κ*B pp65 protein levels were higher (*P* < 0.05) in liver from IUGR lambs, whereas those in liver from IUGR lambs under Arg or NCG treatment were lower than those in IUGR lambs. These findings indicated that supplementing Arg or NCG reduced the contents of proinflammatory cytokines at the same time when the apoptosis-related pathway was being suppressed, thus suppressing the IUGR-induced apoptosis of hepatic cells.

## 1. Introduction

Maternal undernutrition is associated with intrauterine growth retardation (IUGR) which is detrimental to survival, growth, and metabolism and the long-term performance of the offspring in various animals [1–3]. IUGR alters the development of fetal organs and tissues and may negatively affect their functions in adult life with a predisposition to developing metabolic disorders [4–10]. IUGR has been reported to reduce fetal liver growth and disrupt the metabolic, endocrine, and antioxidant defense functions of the liver in fetal, neonatal, postnatal, and adult life stages [11–14]. IUGR could be prevented only by optimum nutrition during different stages of pregnancy. However, under certain circumstances of nutritional insufficiency/fluctuation, IUGR cannot be totally unavoidable. There is a potential to ameliorate the adverse effects of IUGR on postnatal health and performance through early life nutrition interventions with key functional nutrients [15–17].

Maternal nutrition of L-arginine (L-Arg), a semiessential amino acid, and its precursor N-carbamylglutamate (NCG) during pregnancy has been shown to mitigate the IUGR-metabolic disruptions in ovine fetus [18–21] and rat offspring [22] and enhance fetal development and neonatal survival and growth in mammals [23–25]. Also, L-Arg and NCG have been shown to protect the liver against lipid peroxidation [26–28] as well as play a key role in inflammatory response regulation [29]. Our team conducted a series of studies on Hu sheep, a prolific and precocious native breed in China, where L-Arg and NCG were fed to either underfed ewes from day 35 to day 110 of gestation [18, 21, 30, 31] or to IUGR lambs in the period from day 7 to day 28 after birth [32]. Results of these study series revealed the capability of L-Arg and NCG to enhance fetal and placental development and antioxidant capacity [21]; ameliorate fetal growth retardation in underfed ewes [30]; improve the amino acid profile in the liver and muscles; modulate the somatotropic axis gene expression in fetuses from underfed ewes [18]; and improve intestinal integrity and energy balance, immune response, and antioxidant defense of IUGR suckling lambs [32]. Also, results indicated that both L-Arg and NCG alter the major nutrient and oxidative stress metabolic pathways in fetuses of underfed ewes [31].

However, the effect of early nutrition of L-Arg or NCG on hepatic inflammation and apoptosis in IUGR lambs is still unclear. Therefore, the current study is aimed at investigating the effects of dietary supplementation of L-Arg and NCG on hepatocyte inflammation and apoptosis in IUGR suckling lambs.

## 2. Materials and Methods

All trials were conducted in accordance with the law of animal protection approved by the Guide for the Care and Use of Laboratory Animals prepared by the Ethics Committee of Yangzhou University (SXXY 2015-0054).

### 2.1. Animals and Treatments

Lambs were recognized as either with normal body weight (NBW) or IUGR lambs based on their body weight at birth. Lambs whose birth weight is close to the average birth weight (±0.5 SD) were identified as NBW lambs, whereas lambs with at least 1.5 SD lower birth weight were defined as IUGR [33]. Accordingly, 48 lambs weighing 4.25 ± 0.14 kg (NBW) or 3.01 ± 0.13 kg (IUGR) were selected from 432 twin lambs born to Hu ewes at the Jiangyan Experimental Station (Taizhou, Jiangsu, China) on day 7 after birth. On day 7 after birth, lambs were classified, based on their birth weight, into four experimental groups in a randomized complete block design; each group included 12 lambs distributed to 3 replicates, each with 4 lambs (2 males and 2 females) housed into the indoor cages. All groups were fed a milk replacer (MR) provided by the Tianke and Hejia Company (Nanjing, Jiangsu, China) as a basal diet (Table 1) with free access to clean fresh water. The MR was designed based on the nutrient recommendation of suckling lambs, and its feeding level was adjusted at 2% of the live body weight [34]. The experimental groups were (1) the control group (CON)—lambs with NBW and fed MR only; (2) the IUGR group—lambs with IUGR and fed MR only; (3) the L-Arg group—IUGR lambs fed MR plus 1% L-Arg (Ajinomoto Co., Ltd. (Beijing, China)); or (4) the NCG group—IUGR lambs fed MR plus 0.1% NCG of 97% purity provided by Sigma-Aldrich (St. Louis, Missouri, USA). The L-Arg and NCG doses were designed based on previous research [18, 21, 35]. These diets featured the same amounts of nitrogen (equilibrated by alanine) and energy. L-alanine was provided by Ajinomoto Co., Ltd. (Beijing, China). The experiment lasted for 3 weeks. The MR amount was adjusted at 10-day intervals and was offered to lambs over three times a day after dissolving into hot water. The obtained MR solution was of 40°C and 16.67% DM. The intake and refusal of MR were recorded daily, and the daily dry-matter intake was calculated accordingly (daily MR intake multiplied by dry matter %). Lamb body weights were recorded at the start (7 days old) and at the end (28 days old) of the experiment.

### 2.2. Sample Collection

On the last day of the experiment, lambs were anesthetized using sodium pentobarbital (15 mg/kg body weight) before slaughtering. Blood samples were collected into EDTA-containing tubes, centrifuged at 3,000 × *g* for 15 min at 4°C, and plasma was kept at -80°C until the assay of the proinflammatory cytokines as well as the parameters in liver biochemistry. Also, the fresh liver was collected and weighed, rinsed with phosphate-buffered saline (PBS, pH 7.4), cut into small pieces, and finally preserved at -80°C.

### 2.3. Biochemical Parameter Analysis

Plasma samples were analyzed for aspartate aminotransferase (AST, cat. C010-3), alanine aminotransferase (ALT, cat. C009-3), alkaline phosphatase (ALP, cat. A059-1), albumin (ALB, A028-1), and total protein (T-Pro, cat. A045-3) using commercial ELISA kits (Jiancheng Bioengineering Institute, Nanjing, China) and a spectrophotometer (V-5600, Shanghai, China) in accordance with the manufacturer's instructions.

### 2.4. Radioimmunoassay

Using commercial kits and the BioTek Synergy HT Microplate Reader (BioTek Instruments, Winooski, VT, USA) at a wavelength of 450 nm, the proinflammatory cytokines including interleukin 1*β* (IL-1*β*, R&D Systems, Oxford, UK), interleukin 6 (IL-6, BioSource/MED Probe, Camarillo, CA, USA), and tumor necrosis factor *α* (TNF-*α*, R&D Systems, Oxford, UK) were measured in plasma. The detection limits were 30.0, 10.0, and 7.0 pg/mL for IL-1*β*, IL-6, and TNF-*α*, respectively. The inter- and intra-assay coefficients of variation were ≤10%.

### 2.5. Analysis of the Activities of Caspase-3, Caspase-8, and Caspase-9, Together with Mitochondrial Cytochrome C Assay

The activities of caspase-3 (KGA203), caspase-8 (KGA303), and caspase-9 (KGA403) were determined using colorimetry kits (KeyGEN Biotech. Co., Ltd., Nanjing, China). The liver (approximately 0.1 g) was subjected to homogenization and lysis using a 50 mL cold lysis buffer. Subsequently, the cell lysates were clarified by 5 min of centrifugation at 10,000 g for 5 min at 4°C. Later, 50 mL aliquots of supernatants were collected for analysis in accordance with the manufacturer's instruction.

The 10% hepatic homogenate was subject to 10 min of centrifugation at 2000 g, followed by the transfer of supernatants for 15 min of centrifugation at 10,000 g. Afterward, the obtained pellet was resuspended, followed by lysis using cold lysis buffer (1.5 mL) to estimate the cytochrome C according to the colorimetric method using Nanodrop (WFJ 2100, UNIC Instrument Co., Ltd., Shanghai, China). Additionally, a calibration curve was also established with the use of bovine cytochrome C as previously described [36].

### 2.6. Liver Concentrations of DNA, Protein, and Proliferation Index

Frozen liver (0.5 g) was subjected to homogenization into 20 mL buffer (consisting of 0.05 M Na_3_PO_4_, 2.0 M NaCl, and 0.002 M EDTA, pH = 7.4), and the supernatants were utilized to analyze the DNA and protein contents in duplicate. The DNA content was determined using the Hoechst 33,258 (Sigma-Aldrich, B2338, 1 *μ*g mL^−1^) in accordance with the method of Sambrook and Russell [37] using the bovine liver-derived DNA Type I as a reference. The protein content was measured according to the Bradford method using bovine serum albumin (BSA) as a standard [38].

The fresh liver was collected, rinsed by phosphate-buffered saline (PBS, pH 7.4), cut into small pieces, and filtered with the stainless steel 300 mesh. In addition, the liver proliferation index was determined using the PI staining solution (supplemented with 0.25% Triton X-100, 0.5% propidium iodide, and 10 mg mL^−1^ Rnase, 4ABIO, Beijing, China) and expressed as the percentages of cell numbers at phases S, G2, and M in various phases in the cell cycle. The CellQuest software (Becton Dickinson) was employed for data analysis.

### 2.7. Staining the Lamb Liver Histological Sections with Terminal Deoxynucleotidyl Transferase-Mediated Deoxyuridine Triphosphate Nick-End Labeling (TUNEL)

The lamb liver paraffin sections (4 mm in thickness) were deparaffinized, followed by 40 min of proteinase K treatment (50 mg/mL, KeyGEN Biotech. Co., Ltd., Nanjing, China) at 37°C. Then, the sections were washed with PBS for three times, and the fragmented DNA from the apoptotic cells were measured using the TUNEL technique in accordance with the commercial detection kit instructions (KGA7032, KeyGEN Biotech. Co., Ltd., Nanjing, China). In brief, all sections were subjected to 60 min of incubation by the working-strength terminal deoxynucleotidyl transferase (TdT) solution mixed with digoxigenin-deoxyuridine triphosphate (dUTP) and the TdT at 37°C; afterward, the sections were washed with PBS three times, followed by 30 min of incubation with streptavidin-horseradish peroxidase (HRP) at 37°C. Afterward, the sections were washed with PBS for three times, and the TUNEL-positive cells were recognized by diaminobenzidine, followed by hematoxylin counterstaining. The positive and negative controls were set for each case. Then, a standard microscope was used to view the specimens, and 10 high-power fields (HPF, ×400) were selected randomly by 2 observers to measure the total TUNEL-positive cell count. Data were presented in the form of a TUNEL-positive cell count/field as previously described [36].

### 2.8. Gene Expression Detected by Real-Time PCR (RT-PCR)

Total RNA was extracted with the Trizol reagent (Sigma-Aldrich, St. Louis, MO, USA) according to manufacturer's instructions. Then, RT-PCR was performed as described previously [39], using *β*-actin as an internal control gene. Fold changes in target gene mRNA levels relative to *β*-actin were calculated according to the method recommended by Sun et al. [18]. Each experiment was repeated 6 times. The primer sequences (Sangon Biotech, Shanghai, China) were designed by Primer 5.0 and presented in Table 2.

### 2.9. Western Blotting

Total protein was extracted, and the concentration was determined as previously described [32]. Afterward, 50 *μ*g total proteins was mixed with the loading buffer, followed by 5 min of denaturation at 100°C. Later, the protein was separated through 10% sodium dodecyl sulfate-polyacrylamide gel electrophoresis (SDS-PAGE), followed by transfer onto nitrocellulose membranes (BioTrace, Pall Corp., USA). The primary antibodies include NF-*κ*B pp65 (diluted at 1 : 200, cat. 33020; Santa Cruz Biotechnology, CA, USA), NF-*κ*B p65 (diluted at 1 : 200, cat. sc-109, Santa Cruz Biotechnology, CA, USA), caspase-3 (diluted at 1 : 1000, cat. AC030, Beyotime, Shanghai, China), TNF-*α* (diluted at 1 : 200, cat. sc-8301, Santa Cruz Biotechnology, Santa Cruz, CA, USA), Bcl-2 (diluted at 1 : 1000, cat. AB112, Beyotime, Shanghai, China), CtyC (diluted at 1 : 200, cat. AC908, Beyotime, Shanghai, China), Bax (diluted at 1 : 500, cat. AB026, Beyotime, Shanghai, China), Fas (diluted at 1 : 1000, cat. AB067, Beyotime, Shanghai, China), P53 (diluted at 1 : 500, cat. AB118, Beyotime, Shanghai, China), and *β*-actin (diluted at 1 : 5000, cat. AP0060, Bioworld Technology, Inc., St. Louis Park, USA). Afterward, the proteins were further incubated with the HRP-conjugated secondary antibody using the enhanced chemiluminescence solution (cat. P0018F, Beyotime, Shanghai, China). Bands were analyzed by Quantity One Software (Bio-Rad, USA) as previously described [40, 41].

### 2.10. Statistical Analyses

The SPSS16.0 software (SPSS, Chicago, IL, USA) was utilized for statistical analyses. The one-way analysis of variance (ANOVA) was utilized to measure the statistical difference among treatments. Tukey's post hoc test was adopted in multiple comparisons. A difference of *P* < 0.05 was deemed as a statistical significance whereas *P* < 0.1 was declared as a tendency of significance.

## 3. Results

### 3.1. The BW, DNA Concentration, Liver Weight, Protein-to-DNA Ratio, Apoptotic Cell Numbers, and Proliferation Index

The final weight, liver weight, hepatic DNA concentration, protein-to-DNA ratio, and proliferation index were lower (*P* < 0.05), but the hepatic apoptotic cell number in each HPF (×400) was greater (*P* < 0.05) in IUGR lambs compared to CON lambs (Table 3). The dietary supplementation of L-Arg or NCG ameliorated the negative effects of IUGR on the abovementioned parameters but did not restore it to the levels of the CON lambs (*P* < 0.05), and there was no difference between the L-Arg and NCG treatments (*P* > 0.05) (Table 3).

### 3.2. Biochemical Parameter Concentrations

Lambs in the IUGR group had increased ALP, AST, ALT, ALB, and T-Pro in comparison with those of CON lambs (Table 4), while those of the Arg or NCG treatment group were decreased (*P* < 0.05) in comparison with those of the IUGR lambs.

### 3.3. Plasma Proinflammatory Cytokine Concentrations

The plasma TNF-*α*, IL-6, and IL-1*β* contents were higher (*P* < 0.05) in IUGR lambs compared to all treatments (Table 5). The CON lambs showed the lowest values of proinflammatory cytokines (*P* < 0.05). The dietary supplementation of L-Arg or NCG counteracted the adverse effects of IUGR on plasma proinflammatory cytokine concentrations observed in IUGR lambs (*P* < 0.05).

### 3.4. Caspase-3, 8, and 9 Activities, as well as Cytochrome C Level

Livers of IUGR lambs were associated with enhanced (*P* < 0.05) caspase-3, 8, and 9 activities (Table 6), along with an increased cytochrome C level in mitochondria compared with those in the CON group (*P* < 0.05). The activities of caspase-3 and 8 in the liver were reduced (*P* < 0.05) among lambs treated with Arg- or NCG in comparison with those in IUGR lambs. No significant difference (*P* > 0.05) was observed in caspase-9 activity and the cytochrome C level between lambs treated with Arg or NCG and the IUGR lambs.

### 3.5. Relative mRNA Expressions of Selected Inflammatory and Apoptotic Genes

Compared with CON lambs, MyD88; IL-6; TLR-9; IL-1*β*; TLR-4; NF-*κ*B; TNF-*α*; Fas; P53; caspase-3, 8, and 9; and Bax mRNA levels in livers of IUGR lambs were higher (*P* < 0.05) (Table 7), whereas those in lambs treated with Arg or NCG were reduced (*P* < 0.05) in comparison with those in IUGR lambs. Compared with CON lambs, Fasl and Bcl-2 mRNA levels (*P* < 0.05) in livers from IUGR lambs were reduced, whereas those in Arg- or NCG-treated lambs were greater than those in IUGR lambs (*P* < 0.05).

### 3.6. Relative Protein Expression of Selected Inflammatory as well as Apoptotic Genes

Bcl-2 protein expression in liver from IUGR lambs was downregulated compared with that in CON lambs (Figure 1) (*P* < 0.05). Supplementing Arg as well as NCG in the diet of IUGR lambs upregulated Bcl-2 protein level (*P* < 0.05) in livers compared with that in the IUGR group. Compared with CON lambs, relative P53, Fas, Bax, caspase-3, cytochrome C, TNF-*α*, NF-*κ*B p65, and NF-*κ*B pp65 protein levels were higher (*P* < 0.05) in livers from IUGR lambs, whereas those in livers from Arg- or NCG-treated IUGR lambs were lower than those from IUGR lambs (*P* < 0.05).

## 4. Discussion

This study was conducted to address the changes in the growth, proinflammatory cytokines, hepatic inflammatory response, and apoptosis in IUGR suckling lambs supplemented with L-Arg or NCG. In general, the IUGR lambs showed a compromised response in growth, hepatic inflammatory cytokines, and mRNA and protein expressions of selected inflammatory and apoptotic genes. The dietary supplementation of L-Arg and NCG alleviated the adverse effect without a significant difference between L-Arg and NCG. The findings of this study revealed a decrease in the final BW, liver weight, hepatic DNA concentration, protein-to-DNA ratio, and proliferation index with a decrease in the hepatic apoptotic cell number in IUGR suckling lambs compared to the NBW lambs and IUGR lambs supplemented with either L-Arg or NCG. Similar observations (e.g., impaired growth rate and liver weight) were observed in IUGR models of rats, pigs, and lambs [36, 42, 43]. Impaired liver growth in IUGR animal models has been linked to the general retardation in fetal somatic growth [44–47]. The liver growth retardation has been attributed to hepatic cell proliferation suppression, reduction of hepatic cell size reduction caused by the suppressed growth of cells, and/or an increase in hepatic cell loss through apoptosis. The DNA concentration is an index of hyperplasia whereas the protein : DNA ratio is a hypertrophy index [48, 49]. Our data showed a decrease in the DNA content and in the protein : DNA ratio in IUGR suckling lambs that means a decrease in the hyperplasia/hypertrophy index suggesting a decrease in hepatic cell number and size in IUGR suckling lambs as has been previously noticed by Liu et al. [36]. In addition, the liver proliferation index, represented as the percentages of cells at S, G2, and M phases among various cell cycle phases, was reduced indicating a decrease in cell cycle and an increase in hepatic cells arrested at the G1/Go phase in IUGR lambs, which was responsible for the suppressed proliferation of hepatic cells and the retarded liver growth. Previous research indicated that the proliferation of hepatic cells in the fetus was arrested at the G1 phase, and the apoptosis of these cells showed sensitivity to undernutrition-induced IUGR [36]. The adverse effects of IUGR on the above indices of liver growth were mitigated by the L-Arg and NCG supplementation as indicated by improving the protein : DNA ratio.

Previous studies highlighted the protective effect of L-Arg against hepatic cell apoptosis [50, 51] probably through the upregulation of the Bcl-2 antiapoptotic gene [52]. The IUGR-induced increase in hepatocyte apoptosis was further confirmed by the upregulated Bax and Fas expression; enhanced caspase-3, 8, and 9 activities; and elevated mitochondrial cytochrome C level, as well as the downregulated Bcl-2 expression in the liver of IUGR suckling lambs of our study. The apoptosis can be triggered through the extrinsic (the death receptor pathway) or intrinsic (the mitochondrial apoptosis pathway) pathway [53]. Caspases, such as the apoptotic activators (caspase-8 and 9) and the apoptotic executor (caspase-3), have a great effect on implementing the ordered apoptotic process [54]. Furthermore, activating caspases may be a vital link to initiate the apoptotic process [55]. Two pathways are mainly responsible for activating caspases, including the death receptor-regulated pathway (like Fas/FasL) to activate procaspase-8 and the mitochondrion-controlled pathway to activate procaspase-9 [54]. Moreover, the dysfunction of mitochondria, together with the production of mitochondrial cytochrome C in the cytoplasm, marks a vital link to activate caspase-9; subsequently, caspase-3 can implement the apoptotic process [56]; such mechanism is different from that of Bcl-2, the mitochondrial membrane protein [57, 58]. In our study, L-Arg or NCG reversed the Bax, Fas, Bcl-2, caspase-9, caspase-8, and caspase-3 gene levels in IUGR lambs. Similarly, Western blotting validated that L-Arg or NCG effectively regulated the IUGR-induced liver apoptosis. Besides, Arg or NCG evidently suppressed the protein expression of caspase-3, cytochrome C, Bax, and Fas, at the same time when the Bcl-2 in IUGR lambs was being upregulated. Thus, it could be suggested that L-Arg or NCG supplementation attenuated the IUGR-induced hepatic cell apoptosis through promoting Bcl-2 expression as well as the Fas-dependent pathway. P53, a tumor suppressor protein that has been extensively investigated, can regulate apoptosis [59], promote proapoptotic proteins, and inhibit Bcl-2 expression, thereby initiating the caspase cascade to trigger apoptosis [60]. According to our results, P53 was inhibited in IUGR lambs under L-Arg or NCG feeding relative to that in IUGR lambs, suggesting that L-Arg or NCG regulated hepatocyte apoptosis in the IUGR suckling lambs through suppressing the P53 expression.

The liver enzymes (AST, Alt, and ALP), albumin, and total protein were remarkably elevated in IUGR suckling lambs, but this elevation was mitigated upon feeding of L-Arg or NCG to IUGR lambs. The serum activity of AST, ALT, and ALP has been recognized as important indices of liver function. The AST is distributed within the cytoplasm and mitochondria whereas ALT is distributed within the cytoplasm only, and accordingly, the release of both enzymes in circulation implies damage of liver cells. The increased ALP level in serum is related to the impairment of hepatic function resulting from liver cholestasis as well as the destroyed hepatocyte membrane. Similar observations have been reported in low-birth-weight piglets [61]. Also, liver dysfunction has been reported in IUGR infants [62]. Our results suggested that L-Arg or NCG supplementation alleviated the liver function impairment in IUGR lambs as indicated by the decreased serum activity of AST and ALT following Arg or NCG supplementation. The dietary supplementation of L-Arg or NCG has been shown to decrease the AST and ALT levels in rats under oxidative stress [51]. According to our findings, the proinflammatory cytokine concentrations in plasma (IL-1*β*, TNF-*α*, and IL-6) were markedly elevated in IUGR suckling lambs, and the plasma levels showed a consistent trend with their respective gene expression identified in this research. Also, the IUGR upregulated the mRNA expression of MyD88, TLR-4, TRL-9, NF-*κ*B, and TNF-*α* in the liver. These findings confirm the IUGR-induced disruption in hepatic inflammatory and immune responses, and consequently, the IUGR lams may be highly vulnerable to immune and inflammation challenges. A similar disrupted immune response (increased plasma level of IL-1*β* and mRNA expression of TLR-4, IL-1*β*, and NF-*κ*B) was recorded in IUGR piglets [63]. The role of the abovementioned proinflammatory cytokines in the regulation of inflammatory response pathways has been emphasized in previous research [64]. Our findings indicated that the dietary supply of L-Arg or NCG mitigated the IUGR-induced elevation of proinflammatory cytokine levels in plasma and downregulated the IUGR-induced upregulation of IL-1*β*, IL-6, MyD88, TLR-4, TRL-9, NF-*κ*B, and TNF-*α* mRNA expression in the liver. These results indicate that L-Arg and NCG could counteract the liver inflammatory response in IUGR suckling lambs. The anti-inflammatory effect of L-Arg and NCG has been previously described in rat spleen under oxidative stress [65]. The dietary administration of NCG has been shown to alleviate intestinal inflammation in the fish fed Arg-deficient diet [66]. In both studies, L-Arg and NCG modulated the anti-inflammatory response via regulating the mRNA expression of pro- and anti-inflammatory cytokines [65, 66].

To sum up, our results highlighted the IUGR-induced elevation of the circulating proinflammatory cytokines as well as the hepatic mRNA expression of their respective genes. Also, IUGR activated the extrinsic apoptotic pathway which increased the hepatocyte apoptosis. Dietary supplementation of L-Arg or NCG to IUGR lambs decreased the levels of proinflammatory cytokines and suppressed the apoptotic pathway, thus avoiding the IUGR-induced apoptosis of hepatic cells. Our findings could shed more light on controlling hepatic cell apoptosis among IUGR lambs, which can thereby maintain liver health and improve growth performance.

## Figures and Tables

**Table 1:** Ingredient and nutrient composition of milk replacer (dry-matter basis, %).

Component	%
*Ingredients*	
Whey protein concentrate (34% CP)	30.00
Milk fat powder (11% CP)	35.00
Whole milk powder	20.00
a-Casein	5.00
Glucose	6.00
Premix^a^	4.00
Total	100.00
*Nutrient content (analyzed)* ^b^	
GE (MJ/kg)	19.4
CP (%)	23.8
EE (%)	15.7
Ash (%)	7.64
Lysine (%)	1.53
Methionine (%)	0.41
Threonine (%)	0.87
L-arginine (%)	0.63
Ca (%)	0.99
TP (%)	0.73

DM: dry matter; GE: gross energy; CP: crude protein; EE: ether extract; Ca: calcium; TP: total phosphorus. ^a^Main contents of the premixed mixture (per kg of the premixed mixture): Cu (as CuSO_4_·5H_2_O) 600 mg; Mn (MnSO_4_·H_2_O) 315 mg; Fe (FeSO_4_·7H_2_O) 8400 mg; Zn (ZnSO_4_·7H_2_O) 12500 mg; Se (as Na_2_SeO_3_) 17 mg; vitamin A 55000 IU; vitamin E 400 IU; vitamin D 5500 IU; vitamin K 12.5 mg; biotin 2 mg; folacin 7.5 mg; choline 15 mg; riboflavin 100 mg; vitamin B_6_ 175 mg; thiamin 317.5 mg; vitamin B_12_ 500 mg. ^b^Nutrient levels are all measured values.

**Table 2:** The primer sequences used in the real-time PCR.

Genes^a^	Sequences (5′-3′)^b^	Gene bank no.
MyD88	F: ATGGTGGTGGTTGTCTCTGAC R: GGAACTCTTTCTTCATTGGCTTGT	GQ221044
TLR-2	F: CAAGAGGAAGCCCAGGAAG R: TGGACCATGAGGTTCTCCA	DQ890157
TLR-4	F: TGCTGGCTGCAAAAAGTATG R: CCCTGTAGTGAAGGCAGAGC	HQ343416
TLR-9	F: ATGGGCCCCTACTGTG R: CTATTCGGCTGTCGTGG	HQ263217.1
TRAF-6	F: TCAGAGAACAGATGCCCAAT R: GCGTGCCAAGTGATTCCT	XM_012134166.2
IL-6	F: AGGAAAAAGATGGATGCTTCCA R: GACCAGCAGTGGTTTTGATCAA	NM_001009392
IL-1*β*	F: CGTCTTCCTGGGACGTTTTAG R: CTGCGTATGGCTTCTTTAGGG	NM_001009465
NF-*κ*B	F: ATACGTCGGCCGTGTCTAT R: GGAACTGTGATCCGTGTAG	XM_005226864.2
TNF-*α*	F: ACACCATGAGCACCAAAAGC R: AGGCACAAGCAACTTCTGGA	NM_001024860.1
Fas	F: TTTTGCTGTCAGCCTTGTCC R: TGTTCCACTTCTAGCCCATG	NM_001123003.1
Fasl	F: TCTGTGGAGAAGCAAATAGGTC R: AGGGCAATTCCATAGGTGTC	XM_004013705.1
P53	F: CCCGCCTCAGCACCTTAT R: GCACAAACACGCACCTCA	NM_001009403.1
Caspase-3	F: GCTCGAGCTCATGCACATTC R: CCATTGGGCACTTGGCATAC	NM_001286089.1
Caspase-8	F: TTAGCATAGCACGGGAGCAG R: GTCAGCTCATAGATGGGGGC	XM_005676365.1
Caspase-9	F: AGTCAGGCCCTTCCTTTGTT R: ATGGGTCCTGCTTCATCACT	XM_005690814
Bcl-2	F: CGAGTGGCGGCTGAAAT R: GGTCTGCCATGTGGGTGTC	HM630309.1
Bax	F: ATGGGCTGGACATTGGACTT R: ACTGTCTGCCATGTGGGTGT	AF163774.1
*β*-Actin	F: GCTCTTCCAGCCGTCCTT R: TGAAGGTGGTCTCGTGAATGC	NM_001009784.1

*^a^*MyD88: myeloid differentiation factor 88; TRAF-6: TNF receptor-associated factor 6; TLR: Toll-like receptor; IL: interleukin; NF-*κ*B: nuclear factor kappa-B; TNF-*α*: tumor necrosis factor alpha; Bax: Bcl-2-associated X protein; Bcl-2: B-cell lymphoma/leukaemia 2; p53: protein 53; Fas: apoptosis antigen 1; Fasl: Fas ligand. ^b^F: forward; R: reverse.

**Table 3:** Effects of dietary L-arginine and N-carbamylglutamate supplementation on the BW, liver weight, DNA contents, protein : DNA ratio, apoptotic cell numbers, and the proliferation index in the liver of IUGR suckling lambs.

Item	CON	IUGR	IUGR+1%Arg	IUGR+0.1%NCG	*P* value
Initial weight (kg)	4.25 ± 0.11^a^	3.01 ± 0.13^b^	2.9 ± 0.149^b^	3.03 ± 0.10^b^	0.014
Final weight (kg)	7.66 ± 0.28^a^	5.41 ± 0.23^c^	6.01 ± 0.29^b^	6.04 ± 0.30^b^	0.008
Liver weight (g)	172.65 ± 12.13^a^	115.65 ± 12.43^c^	135.78 ± 13.03^b^	137.26 ± 11.17^b^	0.008
Liver weight/BW (%)	2.25 ± 0.06	2.15 ± 0.07	2.26 ± 0.08	2.27 ± 0.09	0.109
DNA contents (mg)	2753 ± 88^a^	1376 ± 91^c^	2008 ± 86^b^	2089 ± 81^b^	0.006
Protein : DNA ratio	12.09 ± 0.67^a^	7.01 ± 0.61^c^	9.98 ± 0.65^b^	10.12 ± 0.71^b^	0.011
Proliferation index (%)	6.96 ± 0.27^a^	3.09 ± 0.35^c^	4.99 ± 0.32^b^	5.01 ± 0.29^b^	0.008
Apoptotic cell per HPF	241 ± 10^c^	367 ± 15^a^	304 ± 11^b^	298 ± 13^b^	0.006

Mean values with their standard errors of the mean (SEM), *n* = 12/group. ^a,b,c^Within a row, means without a common superscript letter differ (*P* < 0.05). HPF: high-power field (×400); CON: the normal birth weight group given a control diet; IUGR: the IUGR group given a control diet; IUGR+Arg: the IUGR group given a L-arginine-supplemented diet; IUGR+NCG: the IUGR group given a N-carbamylglutamate-supplemented diet.

**Table 4:** Effects of dietary L-arginine and N-carbamylglutamate supplementation on biochemical parameter concentrations in the peripheral blood of IUGR suckling lambs.

Item	CON	IUGR	IUGR+1%Arg	IUGR+0.1% NCG	*P* value
AST (IU/L)	30.09 ± 2.10^c^	44.89 ± 2.15^a^	37.12 ± 2.07^b^	36.99 ± 2.18^b^	0.006
ALT (IU/L)	9.13 ± 1.06^c^	18.96 ± 1.17^a^	13.78 ± 1.12^b^	14.01 ± 1.02^b^	0.012
ALP (IU/L)	46.13 ± 3.02^c^	78.12 ± 4.01^a^	60.12 ± 3.66^b^	48.13 ± 3.42^c^	0.007
ALB (g/L)	30.13 ± 2.77^c^	40.24 ± 3.34^a^	36.76 ± 2.89^b^	35.78 ± 2.93^b^	0.009
T-Pro (g/L)	61.43 ± 3.59^c^	79.25 ± 4.07^a^	63.78 ± 3.09^c^	62.13 ± 3.01^c^	0.014

Mean values with their SEM, *n* = 12/group. ^a,b,c^Within a row, means without a common superscript letter differ (*P* < 0.05). AST: aspartate aminotransferase; ALT: alanine aminotransferase; ALP: alkaline phosphatase; ALB: albumin; T-Pro: total proteins; CON: the normal birth weight group given a control diet; IUGR: the IUGR group given a control diet; IUGR+Arg: the IUGR group given a L-arginine-supplemented diet; IUGR+NCG: the IUGR group given a N-carbamylglutamate-supplemented diet.

**Table 5:** Effects of dietary L-arginine and N-carbamylglutamate supplementation on plasma proinflammatory cytokine concentrations from the peripheral blood in the IUGR suckling lambs.

Item	CON	IUGR	IUGR+1%Arg	IUGR+0.1% NCG	*P* value
IL-1*β* (pg/mL)	189.21 ± 17.67^c^	256.38 ± 19.45^a^	218.81 ± 16.33^b^	216.52 ± 17.09^b^	0.008
TNF-*α* (pg/mL)	135.34 ± 9.34^c^	193.23 ± 11.56^a^	159.21 ± 10.11^b^	163.82 ± 12.58^b^	0.015
IL-6 (pg/mL)	151.23 ± 12.35^c^	227.28 ± 16.36^a^	193.55 ± 13.23^b^	190.98 ± 14.16^b^	0.007

Mean values with their SEM, *n* = 12/group. ^a,b,c^Within a row, means without a common superscript letter differ (*P* < 0.05). IL-1*β*: interlukin-1*β*; TNF-*α*: tumor necrosis factor *α*; IL-6: interlukin-6; CON: the normal birth weight group given a control diet; IUGR: the IUGR group given a control diet; IUGR+Arg: the IUGR group given a L-arginine-supplemented diet; IUGR+NCG: the IUGR group given a N-carbamylglutamate-supplemented diet.

**Table 6:** Effects of dietary L-arginine and N-carbamylglutamate supplementation on the caspase-3, caspase-8, and caspase-9 activities, and the levels of cytochrome C in the liver of IUGR suckling lambs.

Item	CON	IUGR	IUGR+1%Arg	IUGR+0.1% NCG	*P* value
Caspase-3	5.36 ± 0.31^c^	10.14 ± 0.56^a^	7.65 ± 0.47^b^	7.59 ± 0.39^b^	0.009
Caspase-8	5.11 ± 0.13^b^	8.56 ± 0.21^a^	5.08 ± 0.07^b^	5.29 ± 0.11^b^	0.015
Caspase-9	4.28 ± 0.21^b^	7.47 ± 0.33^a^	5.74 ± 0.19^ab^	5.86 ± 0.21^ab^	0.022
Cytochrome C (mmol/L)	0.17 ± 0.02^b^	0.24 ± 0.08^a^	0.20 ± 0.05^ab^	0.21 ± 0.03^ab^	0.018

Mean values with their SEM, *n* = 12 in each group. ^a,b,c^Mean values within a row with different superscript letters were significantly different (*P* < 0.05). CON: the normal birth weight group given a control diet; IUGR: the intrauterine growth retardation group given a control diet; IUGR+Arg: the intrauterine growth retardation group given a L-arginine-supplemented diet; IUGR+NCG: the intrauterine growth retardation group given a N-carbamylglutamate-supplemented diet.

**Table 7:** Effects of dietary L-arginine and N-carbamylglutamate supplementation on the relative mRNA expressions of selected inflammatory and apoptotic genes in the liver of IUGR suckling lambs.

Item	CON	IUGR	IUGR+1%Arg	IUGR+0.1% NCG	*P* value
MyD88	1.00 ± 0.15^b^	1.76 ± 0.21^a^	1.03 ± 0.14^b^	0.99 ± 0.11^b^	0.017
TLR-2	1.00 ± 0.05	1.09 ± 0.09	1.02 ± 0.07	1.07 ± 0.04	0.119
TLR-4	1.00 ± 0.05^c^	1.71 ± 0.15^a^	1.39 ± 0.09^b^	1.42 ± 0.14^b^	0.008
TLR-9	1.00 ± 0.06^c^	1.69 ± 0.16^a^	1.33 ± 0.14^b^	1.38 ± 0.16^b^	0.011
TRAF-6	1.00 ± 0.09	0.99 ± 0.11	1.07 ± 0.14	1.03 ± 0.12	0.103
IL-6	1.00 ± 0.07^b^	1.48 ± 0.17^a^	1.03 ± 0.14^b^	0.99 ± 0.08^b^	0.004
IL-1*β*	1.00 ± 0.08^c^	1.76 ± 0.15^a^	1.38 ± 0.11^b^	1.41 ± 0.09^b^	0.010
NF-*κ*B	1.00 ± 0.04^c^	1.73 ± 0.11^a^	1.42*b* ± 0.09	1.41 ± 0.06^b^	0.008
TNF-*α*	1.00 ± 0.07^c^	1.59 ± 0.14^a^	1.32 ± 0.11^b^	1.31 ± 0.13^b^	0.007
Fas	1.00 ± 0.11^c^	2.09 ± 0.21^a^	1.51 ± 0.11^b^	1.57 ± 0.15^b^	0.009
Fasl	1.00 ± 0.05^a^	0.35 ± 0.03^c^	0.67 ± 0.07^b^	0.64 ± 0.06^b^	0.005
P53	1.00 ± 0.08^c^	1.98 ± 0.19^a^	1.49 ± 0.15^b^	1.44 ± 0.11^b^	0.014
Caspase-3	1.00 ± 0.09^c^	2.12 ± 0.17^a^	1.49 ± 0.10^b^	1.47 ± 0.15^b^	0.012
Caspase-8	1.00 ± 0.08^c^	1.86 ± 0.17^a^	1.42 ± 0.10^b^	1.39 ± 0.12^b^	0.007
Caspase-9	1.00 ± 0.11^c^	1.79 ± 0.18^a^	1.37 ± 0.15^b^	1.40 ± 0.12^b^	0.011
Bcl-2	1.00 ± 0.08^a^	0.48 ± 0.05^c^	0.73 ± 0.06^b^	0.71 ± 0.09^b^	0.008
Bax	1.00 ± 0.12^c^	2.56 ± 0.18^a^	1.79 ± 0.11^b^	1.71 ± 0.13^b^	0.006

Mean values with their SEM, *n* = 12 in each group. ^a,b,c^Mean values within a row with different superscript letters were significantly different (*P* < 0.05). MyD88: myeloid differentiation factor 88; TRAF-6: tumor necrosis factor receptor-associated factor 6; TLR: Toll-like receptor; IL: interleukin; TNF-*α*: tumor necrosis factor *α*; p53: protein 53; Bax: Bcl-2-associated X protein; Bcl-2: B-cell lymphoma 2; Fas: apoptosis antigen 1; Fasl: Fas ligand; CON: the normal birth weight group given a control diet; IUGR: the intrauterine growth retardation group given a control diet; IUGR+Arg: the intrauterine growth retardation group given a L-arginine-supplemented diet; IUGR+NCG: the intrauterine growth retardation group given a N-carbamylglutamate-supplemented diet.

## Data Availability

The scientific and statistical data used to support the findings of this study are included in the article. Requests for access to these data should be addressed to Honghua Jiang at jianghonghua502@126.com.

## Supplementary Materials

Supplementary Materials Figure 1: effects of dietary L-arginine and N-carbamylglutamate supplementation on the relative protein expressions of selected inflammatory and apoptotic genes in the liver of IUGR suckling lambs. P53 (A), Fas (B), Bax (C), Bcl-2 (D), caspase-3 (E), cytochrome C (F), TNF-*α* (G), NF-*κ*B p65 (H), and NF-*κ*B pp65 (I) were determined. Values are means, with standard errors represented by vertical bars. The protein expression value = densitometry units of selected protein/densitometry units of *β* − actin detected by Western blotting. p53: protein 53; Bax: Bcl-2-associated X protein; Bcl-2: B-cell lymphoma 2; Fas: apoptosis antigen 1; TNF-*α*: tumor necrosis factor *α*; NF-*κ*B: nuclear factor kappa-B; CON: the normal birth weight group given a control diet; IUGR: the intrauterine growth retardation group given a control diet; IUGR+Arg: the intrauterine growth retardation group given an arginine-supplemented diet; IUGR+NCG: the intrauterine growth retardation group given a N-carbamylglutamate-supplemented diet. *n* = 12/group. Mean values in columns with unlike superscript letters were significantly different (*P* < 0.05).
