# Supplementary material for: Dietary Supplementation of L-Arginine and N-Carbamylglutamate Attenuated the Hepatic Inflammatory Response and Apoptosis in Suckling Lambs with Intrauterine Growth Retardation
Source: Mediators Inflamm. 2020 Apr 4;2020:2453537. doi: 10.1155/2020/2453537 (PMC7160735; doi:10.1155/2020/2453537)
Supplement: Supplementary Materials — Figure 1: effects of dietary L-arginine and N-carbamylglutamate supplementation on the relative protein expressions of selected inflammatory and apoptotic genes in the liver of IUGR suckling lambs. P53 (A), Fas (B), Bax (C), Bcl-2 (D), caspase-3 (E), cytochrome C (F), TNF-α (G), NF-κB p65 (H), and NF-κB pp65 (I) were determined. Values are means, with standard errors represented by vertical bars. The protein expression value = densitometry units of selected protein/densitometry units of β − actin detected by Western blotting. p53: protein 53; Bax: Bcl-2-associated X protein; Bcl-2: B-cell lymphoma 2; Fas: apoptosis antigen 1; TNF-α: tumor necrosis factor α; NF-κB: nuclear factor kappa-B; CON: the normal birth weight group given a control diet; IUGR: the intrauterine growth retardation group given a control diet; IUGR+Arg: the intrauterine growth retardation group given an arginine-supplemented diet; IUGR+NCG: the intrauterine growth retardation group given a N-carbamylglutamate-supplemented diet. n = 12/group. Mean values in columns with unlike superscript letters were significantly different (P < 0.05). [file 2453537.f1.pdf]

The supplementary material

FIGURE 1: Effects of dietary L-arginine and N-carbamylglutamate supplementation on the relative protein expressions of selected inflammatory and apoptotic genes in the liver of IUGR suckling lambs. P53 (A), Fas (B), Bax (C), Bcl2 (D), caspase 3 (E), cytochrome C (F)，TNF-α (G), NF-κB p65 (H), and NF-κB pp65 (I) were determined. Values are means, with standard errors represented by vertical bars. The protein expression value = densitometry units of selected protein/densitometry units of β-actin detected by Western blotting. p53, protein 53; Bax, Bcl-2 associated X protein; Bcl-2, B-cell lymphoma 2; Fas, apoptosis antigen 1; TNF-α, tumour necrosis factor α; NF-κB, nuclear factor kappa-B; CON, normal birth weight group given a control diet; IUGR, intrauterine growth retardation group given a control diet; IUGR+Arg, intrauterine growth retardation group given a arginine-supplemented diet; IUGR+NCG, intrauterine growth retardation group given a N-carbamylglutamate-supplemented diet. n=12/group. Mean values in columns with unlike superscript letters were significantly different ($P < 0.05$).

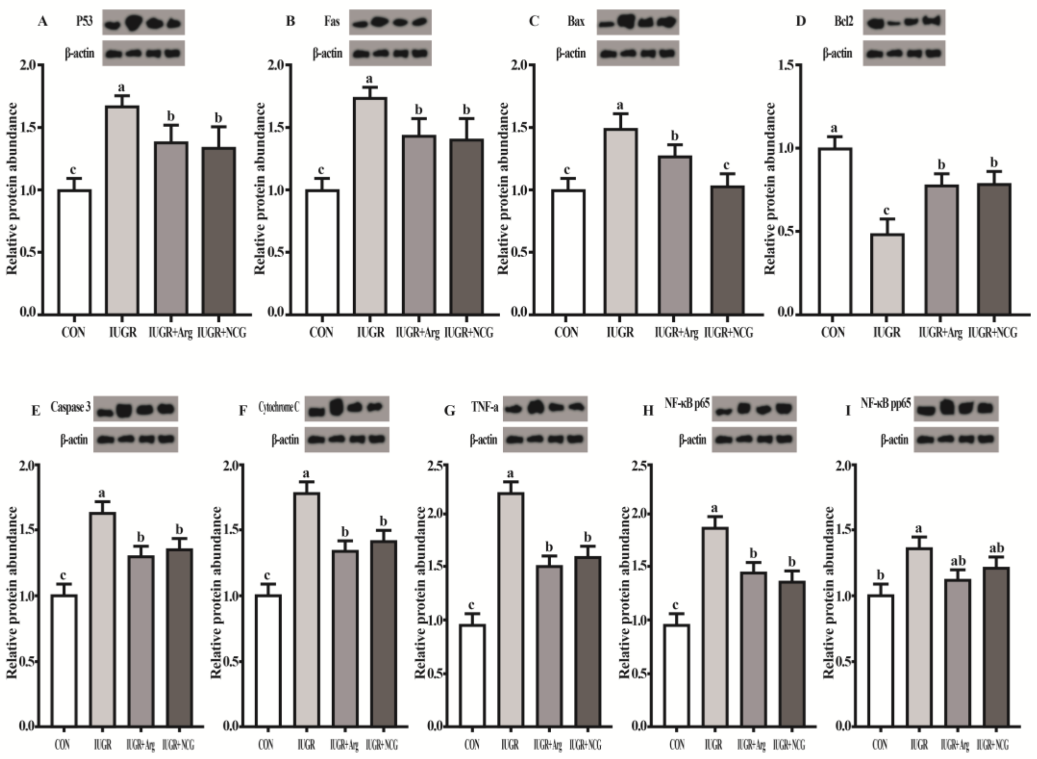
A
P53
β-actin
B
Fas
β-actin
C
Bax
β-actin
D
Bcl2
β-actin
Relative protein abundance
CON
IUGR
IUGR+Arg
IUGR+NCG
E
Caspase 3
β-actin
F
Cytochrome C
β-actin
G
TNF-a
β-actin
H
NF-κB p65
β-actin
I
NF-κB pp65
β-actin
